# Evaluation of safety and efficacy of autologous oral mucosa-derived epithelial cell sheet transplantation for prevention of anastomotic restenosis in congenital esophageal atresia and congenital esophageal stenosis

**DOI:** 10.1186/s13287-023-03321-8

**Published:** 2023-04-13

**Authors:** Akihiro Fujino, Yasushi Fuchimoto, Teizaburo Mori, Motohiro Kano, Yohei Yamada, Michinobu Ohno, Yoshiyuki Baba, Nobutaka Isogawa, Katsuhiro Arai, Takako Yoshioka, Makoto Abe, Nobuo Kanai, Ryo Takagi, Masanori Maeda, Akihiro Umezawa

**Affiliations:** 1 Division of Surgery, Department of Surgical Specialties, National Center for Child Health and Development, Tokyo, 157-8535 Japan; 2 Department of Pediatric Surgery, International University of Health and Welfare School of Medicine, 852, Hatakeda, Narita, Chiba 286-8686 Japan; 3 Division of Pedodontics and Orthodontics, Department of Surgical Specialties, National Center for Child Health and Development, Tokyo, 157-8535 Japan; 4 Division of Gastroenterology, Department of Medical Subspecialties, National Center for Child Health and Development, Tokyo, 157-8535 Japan; 5 Department of Pathology, National Center for Child Health and Development, Tokyo, 157-8535 Japan; 6 Kasaoka Division, Department of General Medicine, Okayama University Graduate School of Medicine, Dentistry and Pharmaceutical Sciences, Okayama, 700-8558 Japan; 7 Healthy Aging Innovation Center, Tokyo Metropolitan Geriatric Hospital and Institute of Gerontology, Tokyo, 173-0015 Japan; 8 Institute of Advanced Biomedical Engineering and Science, Tokyo Women’s Medical University, Tokyo, 162-8666 Japan; 9 Center for Regenerative Medicine, National Center for Child Health and Development Research Institute, Tokyo, 157-8535 Japan; 10 MakeWay LLC, Saitama, 350-0461 Japan

**Keywords:** Congenital esophageal atresia, Congenital esophageal stenosis, Anastomotic stenosis, Epithelial cell sheet, Cell sheet transplantation, Somatic stem cells, Regenerative therapy, Endoscopy

## Abstract

**Background:**

We performed the first autologous oral mucosa-derived epithelial cell sheet transplantation therapy in a patient with refractory postoperative anastomotic stricture in congenital esophageal atresia (CEA) and confirmed its safety. In this study, patients with CEA and congenital esophageal stenosis were newly added as subjects to further evaluate the safety and efficacy of cell sheet transplantation therapy.

**Methods:**

Epithelial cell sheets were prepared from the oral mucosa of the subjects and transplanted into esophageal tears created by endoscopic balloon dilatation (EBD). The safety of the cell sheets was confirmed by quality control testing, and the safety of the transplantation treatment was confirmed by 48-week follow-up examinations.

**Results:**

Subject 1 had a stenosis resected because the frequency of EBD did not decrease after the second transplantation. Histopathological examination of the resected stenosis revealed marked thickening of the submucosal layer. Subjects 2 and 3 did not require EBD for 48 weeks after transplantation, during which time they were able to maintain a normal diet by mouth.

**Conclusions:**

Subjects 2 and 3 were free of EBD for a long period of time after transplantation, confirming that cell sheet transplantation therapy is clearly effective in some cases. In the future, it is necessary to study more cases; develop new technologies such as an objective index to evaluate the efficacy of cell sheet transplantation therapy and a device to achieve more accurate transplantation; identify cases in which the current therapy is effective; and find the optimal timing of transplantation; and clarify the mechanism by which the current therapy improves stenosis.

*Trial registration:* UMIN, UMIN000034566, registered 19 October 2018, https://upload.umin.ac.jp/cgi-open-bin/ctr_e/ctr_view.cgi?recptno=R000039393.

**Supplementary Information:**

The online version contains supplementary material available at 10.1186/s13287-023-03321-8.

## Background

Postoperative anastomotic stenosis in congenital esophageal atresia (CEA) and congenital esophageal stenosis (CES) has been reported to occur in 30% to 50% of cases [1–3]. The main symptom of anastomotic stenosis is dysphagia, resulting in feeding difficulties such as choking on food or difficulty in swallowing saliva. Some of these patients are treated with endoscopic balloon dilatation (EBD) with or without local steroid injections and gradually improve. However, there are many cases of refractory anastomotic stenosis that require repeated EBD. These patients not only suffer physical effects such as malnutrition and poor growth, but also often lose the opportunity to enjoy meals with others. In addition, EBD requires hospitalization, which is time-consuming, physically restricting, and emotionally distressing. For patients who require ongoing treatment for restenosis, resection and re-anastomosis of the stenosis or placement of an absorbable stent are recommended [1]. Re-anastomosis is chosen in severe intractable cases. However, in those cases, the upper and lower esophagus had been stitched together and exhibited severe stretching in the initial anastomosis, resulting in suture failure and fibrotic scar stenosis, or anastomosis that requires a substitute esophagus. Re-anastomosis itself could be a life-threatening risk. Furthermore, re-stenosis may occur even after re-anastomosis, so a safer and minimally invasive treatment is desirable. Even if restenosis occurs to some extent, but the lumen of the stenosis is kept at a certain size and/or the stenosis is flexible, there is a possibility that the patient may keep eating and drinking orally. From this point of view, treatment to at least alleviate restenosis is desired.

To improve this situation for such patients, a new regenerative therapy using somatic stem cells was devised, in which autologous oral mucosa-derived epithelial cell sheets prepared from the patient's oral mucosa were transplanted into the laceration site after EBD [4]. This is based on "cell sheet engineering" proposed by Okano et al. at the Institute of Advanced Biomedical Engineering and Science (ABMES), Tokyo Women's Medical University. Endoscopic submucosal dissection (ESD) is performed for superficial esophageal squamous cell carcinoma (ESCC) in adults, but extensive dissection of the esophageal mucosa and submucosa can cause esophageal stenosis after surgery. Okano et al. reported that transplantation of autologous oral mucosa-derived epithelial cell sheets into ulcerated areas after ESD effectively prevented esophageal stenosis [5–7]. Based on these previous studies, we confirmed the efficacy of cell sheet transplantation in preventing restenosis by performing cell sheet transplantation after EBD in a porcine model of esophageal stricture as a preclinical study in order to apply cell sheet transplantation to refractory anastomotic stricture after surgery for congenital esophageal atresia [8]. Then, we conducted the first clinical trial in humans [4]. Autologous oral mucosa-derived cell sheets were produced from the subject's oral epithelial tissue and transplanted into the laceration site after EBD using a newly developed pediatric transplantation device. The safety of this treatment in humans was confirmed by quality control testing of the cell sheets and follow-up examinations for 48 weeks after transplantation. In this study, the safety and efficacy of cell sheet transplantation therapy for refractory esophageal anastomotic stenosis were evaluated in additional subjects.

## Methods

### Subject information

This study included patients between 1 and 30 years of age with postoperative anastomotic restenosis of CEA and CES who had repeated restenosis after at least 5 balloon dilatations. Three subjects have been treated so far (Table 1).

**Table 1:** Information on subjects

	Subject 1	Subject 2	Subject 3
Age at transplant	16Y	19Y	13Y
Sex	Male	Male	Male
Height (cm) just before transplantation	165.3	154.7	154.9
Weight (kg) just before transplantation	43.7	38.2	43.0
Diagnosis	Esophageal atresia (Type B)	Esophageal atresia (Type A)	Esophageal stenosis
EBD frequency before transplant	Every 2 or 3 weeks	Every 3 months	2 or 3 times a year

### Fabrication of oral mucosal epithelial cell sheets

In accordance with our previous study, cultured autologous oral mucosal epithelial cell sheets were fabricated by using buccal mucosal tissue and serum derived from the patient at the cell processing facility (CPF) of CellSeed Inc. [4]. After the cultivation for 16 days, the epithelial cells were transported to the National Center for Child Health and Development (NCCHD) from CPF of CellSeed at 37 °C and transplanted on wounded esophageal mucosa in the operating room right after EBD (Additional file 1: Table S1).

### Quality control tests

In accordance with our previous study, quality control tests were carried out by CellSeed Inc. before transplantation of cultured autologous oral mucosal epithelial cell sheets [4]. Quantification for cellular density, viability, and percentage of epithelial cells in the epithelial cell sheets was also implemented.

### Endoscopic balloon dilatation (EBD) for anastomotic stenosis

EBD was performed on each subject as in the first transplantation on subject 1 [4]. In subject 1, the balloon size was increased to 18 mm, 19 mm, and 20 mm and kept dilated for 180 s each time. A 13.5-mm balloon and 3 dilations of 180 s were used for subject 2. A 20-mm balloon and 3 dilations of 180 s were used for subject 3.

### Endoscopic cell sheet transplantation using a dedicated transplant device

Cell sheets were transplanted into each subject under the same conditions as for the first transplantation in subject 1 [4]. Subject 1 was transplanted with ESC-002 (sheet ID), subject 2 with ESC-003, and subject 3 with ESC-004 (Additional file 1: Table S1).

### Follow-up examination

To assess safety and efficacy, scheduled follow-up examinations were performed for 48 weeks after transplantation of cell sheets in each subject under the same conditions as in the first transplantation in subject 1 (Additional file 2: Table S2) [4].

## Results

### Fabrication of epithelial cell sheets

In all cases, epithelial cell sheets were fabricated in accordance with standard operating procedures (SOPs) and passed quality control testing (Additional file 1: Table S1, Additional file 3: Table S3, Additional file 4: Fig. S1).

### Transplantation of epithelial cell sheets and progress in subject 1

**Fig. 1:**
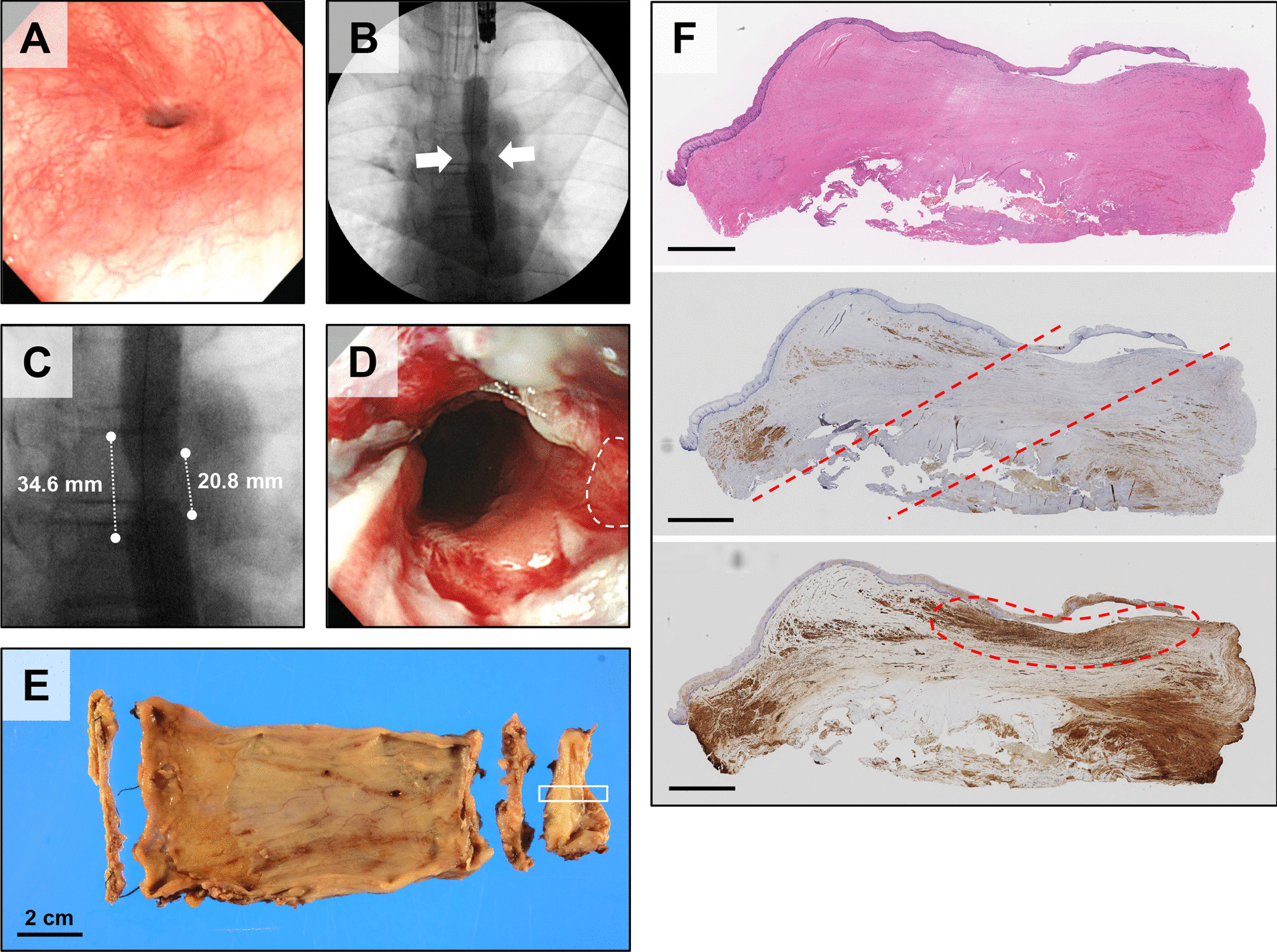
Second epithelial cell sheet transplantation in subject 1. **A** Endoscopic image of the transplanted area approximately 3 weeks (23 days) after the first epithelial cell sheet transplantation. The first EBD after transplantation was performed on the same day. **B** Contrast esophagography during EBD. Arrows indicate the stenotic area. The balloon was filled with contrast at 1 atm of internal pressure. **C** Enlarged image of the esophageal stenosis. The dotted line on the left side is approximately 34.6 mm, and the right side is 20.8 mm. **D** Endoscopic image immediately after the second cell sheet transplantation. The cell sheet was transplanted in the mucosal defect area indicated by the dotted line. **E** Macroscopic view of the resected postoperative anastomotic stenosis of esophageal atresia (right: mouth side, left: stomach side). **F** Histology of the stenotic region surrounded by the white line in Fig. 1E. The submucosal layer was thickened with fibrotic tissue (HE staining, upper panel). In the area between the dotted lines, a lack of continuity of the muscle layer was observed (desmin staining, middle panel). In the area enclosed by the dotted line, an accumulation of myofibroblasts was observed in the submucosa of the smooth muscle defect (*α*-SMA staining, lower panel). Scale bar is 2 mm

A 16-year-old male with postoperative anastomotic stenosis of esophageal atresia type B was the first subject for whom a second epithelial cell sheet transplantation was performed (Table 1, Fig. 1). Subject 1 had restenosis after the first cell sheet transplantation and underwent EBDs (Fig. 1A). Since EBD was required every 2 to 4 weeks thereafter, a second transplant was performed 13 months after the first transplant. The length of the stenosis was measured on contrast-enhanced images as 20.8 mm to 36.4 mm, depending on the site (Fig. 1B, C). The EBD before transplantation resulted in a circumferential laceration, a longitudinal laceration extending the entire length of the stenosis at a side, and detachment of the esophageal mucosa (Fig. 1D). Four of the cell sheets (ESC-002) were used for transplantation (Additional file 1: Table S1). As seen after the first transplantation, the subject experienced improvement in swallowing food and drink without blockage for some time after the second transplantation. However, the frequency of EBD returned quickly to the pre-transplantation level, and surgical resection of the stenosis was performed 4 months after the second transplantation. Histopathological examination of the resected esophageal stenotic site revealed prominent fibrosis and thickening of the submucosa (HE staining) (Fig. 1E, F). The thickness of the submucosal layer was 1.8 to 2.0 mm. Desmin staining and alpha-smooth muscle actin (*α*-SMA) staining revealed a lack of muscular continuity in the stenotic site. An accumulation of myofibroblasts was observed in the submucosa of the area of smooth muscle defect.

### Transplantation of epithelial cell sheets and progress in subject 2

**Fig. 2:**
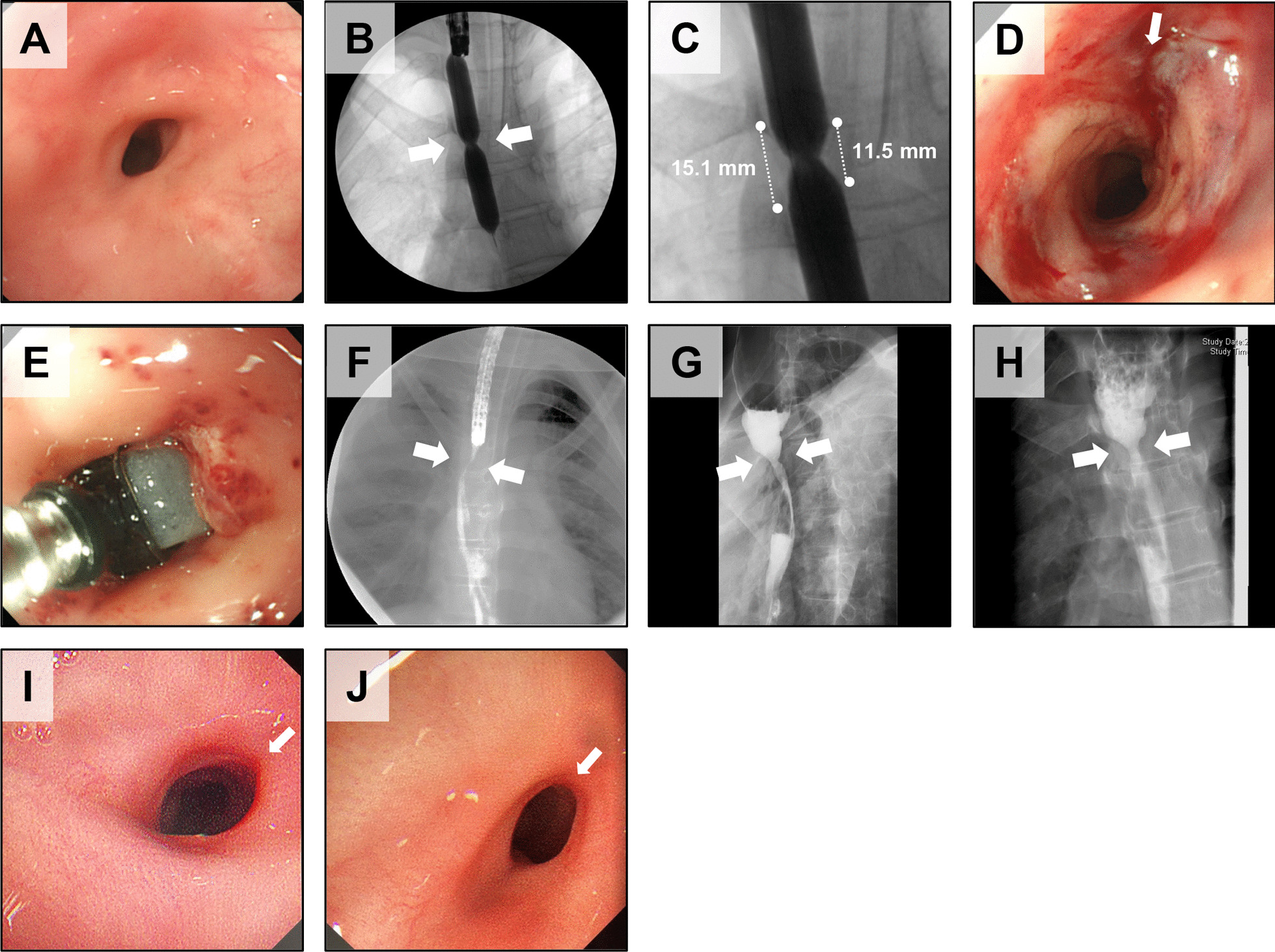
Epithelial cell sheet transplantation into subject 2. **A** Endoscopic image of the stenosis just before EBD at cell sheet transplantation. **B** Contrast esophagography during EBD just before cell sheet transplantation. Arrows indicate the stenosis. The balloon was filled with contrast at 1 atm of internal pressure. **C** Enlarged image of the esophageal stenosis. The dotted line on the left is approximately 15.1 mm, and the right is 11.5 mm. **D** Endoscopic image after EBD just before cell sheet transplantation. Arrows indicate the location of the laceration caused by EBD. **E** The cell sheets were applied to the mucosa dehiscence above the laceration using the transplantation device. **F** Contrast esophagography before EBD at cell sheet transplantation. Arrows indicate anastomotic stenosis. **G** Contrast esophagography approximately one month (39 days) after cell sheet transplantation. Arrows indicate the stenosis. **H** Contrast esophagography approximately 5 months (154 days) after cell sheet transplantation. Arrows indicate the stenosis. **I** Endoscopic image of the stenosis approximately 5 months (154 days) after cell sheet transplantation. Arrows indicate the location of the laceration caused by EBD immediately before transplantation. **J** Endoscopic image of the stenosis approximately 12 months (348 days) after cell sheet transplantation. Arrows indicate the location of the laceration caused by EBD immediately before transplantation

The second subject was a 19-year-old male with postoperative anastomotic stenosis of esophageal atresia type A (Table 1, Fig. 2). The length of the stenosis was measured on contrast-enhanced images as 11.5 mm to 15.1 mm, depending on the site (Fig. 2B, C). The laceration caused by EBD at transplantation was localized to a single site of laceration over the entire length of the stenosis and a detachment of the esophageal mucosa in the vicinity (Fig. 2D). Three cell sheets (ESC-003) were transplanted (Additional file 1: Table S1, Fig. 2E). Comparison of the diameter at the esophageal stricture in pre- and post-transplantation contrast esophagographies showed no noticeable change (Fig. 2F–H). Endoscopic examination at 1, 3, and 6 months after transplantation revealed that the folds behind the stenosis were more easily visible than before transplantation, and the stenosis was very soft (Fig. 2I, J). The subject experienced a clear improvement in swallowing food and drink without blockage after transplantation. Before transplantation, the subject was only able to consume a paste diet 3 months after EBD, but he was able to consume a normal diet after transplantation.

### Transplantation of epithelial cell sheets and progress in subject 3

**Fig. 3:**
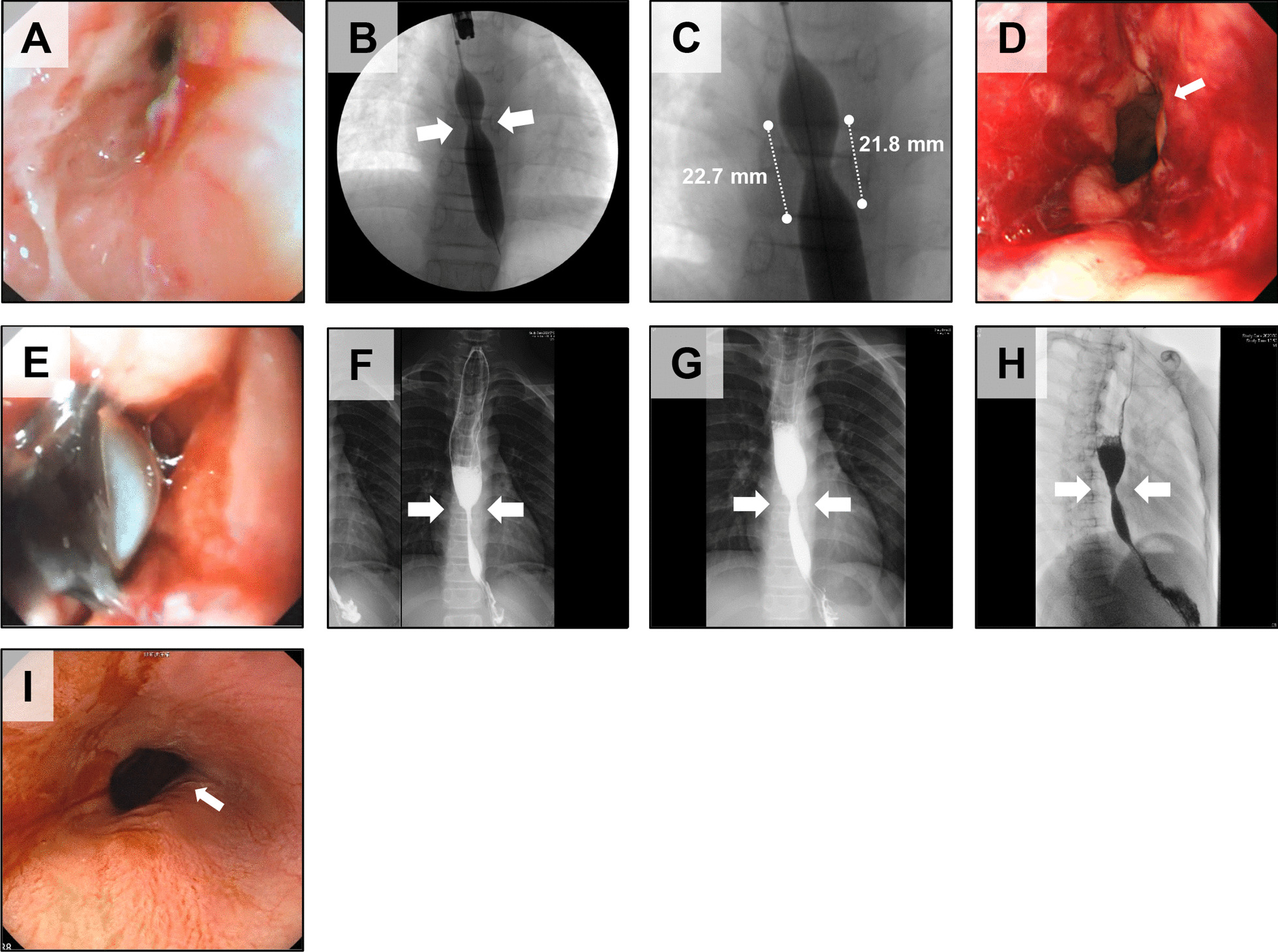
Epithelial cell sheet transplantation into subject 3. **A** Endoscopic image of the stenosis just before EBD at cell sheet transplantation. **B** Contrast esophagography during balloon dilation just before cell sheet transplantation. Arrows indicate the stenosis. The balloon was filled with contrast at 1 atm of internal pressure. **C** Enlarged image of the esophageal stenosis. The dotted line on the left is approximately 22.7 mm and the right is 21.8 mm. **D** Endoscopic image after balloon dilation just before cell sheet implantation. Arrows indicate the location of the laceration caused by balloon dilation. **E** The cell sheets were attached to the mucous dehiscence above the laceration using the transplantation device. **F** Contrast esophagography approximately one month before cell sheet transplantation. Arrows indicate anastomotic stenosis. **G** Contrast esophagography approximately 3 months (91 days) after cell sheet transplantation. Arrows indicate stenosis. **H** Contrast esophagography approximately five and a half months (166 days) after cell sheet transplantation. Arrows indicate the stenosis. **I** Endoscopic image of the stenosis approximately one month (28 days) after cell sheet transplantation. Arrows indicate the location of the laceration caused by balloon dilation just before transplantation

The third subject was a 13-year-old male with postoperative anastomotic stenosis of a congenital esophageal stenosis (Table 1, Fig. 3). The length of the stenosis was measured on contrast-enhanced images as 21.8 mm to 22.7 mm depending on the site (Fig. 3B, C). The EBD at transplantation caused a circumferential laceration, a longitudinal laceration extending the entire length of the stenosis on one side, and detachment of the esophageal mucosa (Fig. 3D). Three cell sheets (ESC-004) were transplanted (Additional file 1: Table S1, Fig. 3E). Comparison of the diameter at the esophageal stricture in pre- and post-transplantation contrast esophagographies showed no noticeable change, although the diameter of the stricture appeared to be slightly larger after transplantation (Fig. 3F–I). The subject experienced an improvement in swallowing food and drink without blockage after transplantation. The time required for a single meal was reduced from 2 h to about 30 min.

### EBDs before and after epithelial cell sheet transplantation

**Fig. 4:**
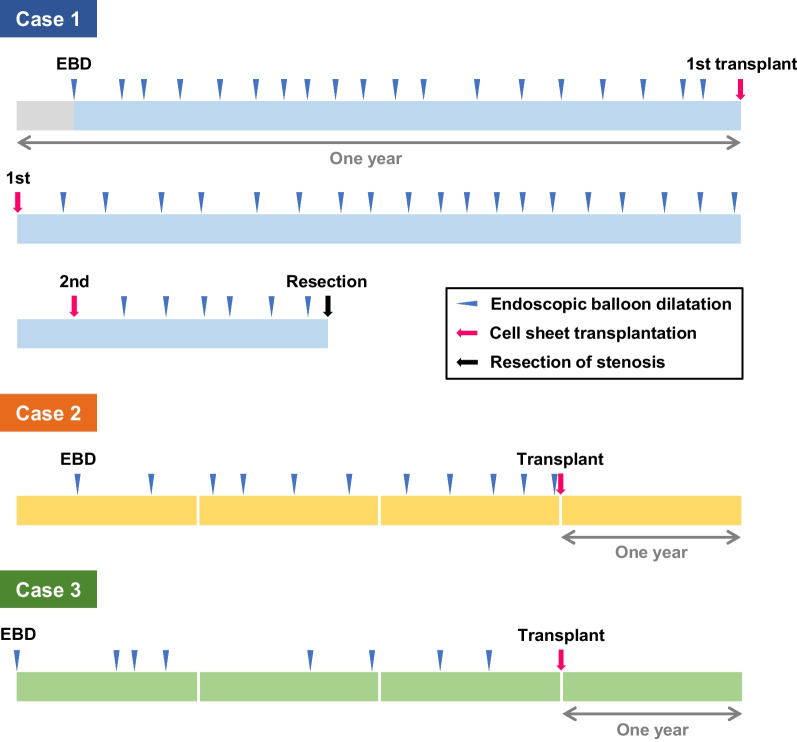
Endoscopic balloon dilatation before and after cell sheet transplantation in each case. The time axis shows the status of EBD before and after cell sheet transplantation in subjects 1–3. Blue triangles indicate EBD, red arrows indicate cell sheet transplantation, and a black arrow indicates resection of the stenosis. The distance between triangles and arrows indicates the interval between procedures

The time course of EBD before and after cell sheet transplantation for each subject is shown in Fig. 4. In case 1, EBDs were performed less frequently for 6 months after the first transplantation but returned to the same level of frequency as before transplantation; after the second transplantation, the frequency of EBD did not decrease, and the stenosis was finally resected surgically. In case 2, EBD was performed every 3 months before transplantation, but not for 48 weeks after transplantation. In case 3, EBD was performed 2–3 times a year before transplantation, but as in case 2, it was not performed for 48 weeks after transplantation.

### Confirmation of safety

Appropriate follow-up examinations were performed on all subjects. All physical and laboratory tests showed no abnormalities, confirming the safety of the transplant.

## Discussion

### Effect of cell sheet transplantation for the postanastomotic stenosis of CEA and CES

Subject 1 underwent two transplantations and showed temporary improvement of symptoms immediately after each transplantation, but the stenosis site was eventually resected. The cell sheet transplantation had a limited effect on subject 1. In the resected esophageal stenosis area, fibrosis of the submucosal layer was noticeable, and the submucosal layer was thickened. The thickness of the submucosa ranged from 1.8 to 2.0 mm, which is more pronounced than the average thickness in the normal esophagus (0.17 mm to 0.24 mm). This suggests that repeated damage and repair caused by EBD may have stimulated inflammation, leading to severe intractable stricture with thickening of the esophageal wall. Subjects 2 and 3, in contrast, showed a clear benefit from cell sheet transplantation treatment. This is because the subjects themselves felt an improvement in food and drink blockage without the need for EBD during the 48-week follow-up period after transplantation. In addition, the improvement in the passage of food and drink despite no significant change in the size of the esophageal lumen before and after transplantation suggests that the esophageal tissue in the stenotic area gained flexibility and expanded when food and drink passed through the stenosis. These results suggest that flexibility of the stenosis site may become a new index for evaluating treatment efficacy in the future.

### Differences in the effects of cell sheet transplantation between subjects

Comparing the three cases in terms of scarring of esophageal tissue, it is thought that subject 1 had more advanced scarring of esophageal tissue than subjects 2 and 3. This is because subject 1 had undergone more than 100 EBD procedures prior to transplantation, which is an unusually large number, and the repeated dilatation and healing of the esophagus by EBD is assumed to have resulted in the accumulation of inflammatory stimuli. The accumulation of *α*-SMA-positive myofibroblasts in the stenotic area of subject 1 showed evidence of active regenerative scar formation. In addition, subject 1 had a larger stenosis in the longitudinal direction, and the esophageal mucosa was detached entirely circumferentially during dilation, suggesting that the anastomotic stenosis was more extensive than in subjects 2 and 3. It appears that the stenosis in subject 1 was more severe than in subjects 2 and 3. This may have affected the effectiveness of cell sheet transplantation. Conversely, there is a high possibility that restenosis can be prevented if cell sheet transplantation can be performed before the cumulative number of EBD procedures is low, as was the case in cases 2 and 3. Therefore, it is necessary to consider the effective timing and conditions of this treatment.

### Difference of the effect of cell sheet transplantation between ECSS, CEA, and CES

The cell sheet transplant treatment for the three subjects in this study was not as clearly effective as the transplant treatment for ESCC patients. One reason for this may be the thickness of the fibrous scar of the esophageal wall at the transplant site. The treatment of adult ESCC by ESD in the previous study by Okano et al. involved dissection of the esophageal mucosa alone or down to the submucosa. In contrast, in the initial surgical treatment of CEA and CES, the subject of this study, all layers of the esophageal wall were incised and sutured. Then, EBD was repeatedly performed to dilate all layers of the stenotic esophagus after anastomosis. Therefore, the depth of damage to the esophageal wall at the time of transplantation is expected to be much deeper than in patients with ECSS. Significant fibrosis and thickening of the esophageal tissue at the stenosis was also observed in a previous study [9]. In addition, smooth muscle rupture and accumulation of myofibroblasts in the submucosa of the ruptured area were observed in the stenotic area of subject 1. The severity of this scarring may have reduced the effectiveness of the transplantation therapy. Conversely, the fact that the efficacy of cell sheet transplantation therapy was confirmed in severe fibrotic scarring in CEA and CES is considered a significant achievement.

### Improvements in transplantation devices and methods

Further improvement of transplantation devices and techniques for pediatric patients is needed, since CEA and CES patients are children and generally have a narrow esophageal lumen that is difficult to keep open even immediately after EBD. In addition, the condition of the transplant site must be improved to address problems such as edema of the esophagus and bleeding from the lacerated wound after EBD. In order to improve the compatibility of the transplanted sheets, it is necessary to precisely place the cell sheets at the target site so that they do not overlap each other, but the limited working space may be a factor that prevents the cell sheet transplant from being fully effective. Therefore, it is necessary to develop a device that enables accurate transplantation even in very confined spaces, or some new device to keep the esophageal lumen dilated during surgery. We are currently studying ways to address this issue.

### Safety of cell sheet transplantation

Cell sheet transplantation therapy for CEA and CES appears to be a safe and apparently less risky treatment compared to surgical removal of the stenotic esophagus since none of the 3 subjects showed any problems in physical or laboratory examinations, and because the epithelial sheets had passed quality control.

## Conclusions

The course of events after cell sheet transplant treatment differed among the three subjects, but all showed improved food and drink passage after transplantation. The second and third subjects were able to go through the follow-up period, approximately one year after transplantation, without EBD, especially subject 2, whose recovery period is about to reach two years. These results confirm that cell sheet transplantation therapy is effective in some cases. Until now, essentially the only treatment for intractable anastomotic stenosis has been surgical resection, a procedure that is extremely burdensome for the patient and does not prevent stenosis completely. Under such circumstances, the confirmation of the effectiveness of this new, highly safe, and minimally invasive treatment is a breakthrough. We will continue to increase the number of subjects and examine objective indicators for evaluating the effectiveness of this therapy as well as effective timing and conditions for administering the therapy. At the same time, we will improve the transplant device and transplant technique to enhance the effectiveness of the therapy. In addition, it is necessary to clarify the mechanism by which the cell sheet acts on the esophageal tissue in the stenotic area in order to improve the effectiveness of the transplantation.

## Supplementary Information

**Additional file 1: Table S1. **Preparation of oral mucosal epithelial cell sheets.

**Additional file 2: Table S2**. Inspection items for epithelial cell sheet transplantation and their timing.

**Additional file 3: Table S3**. Quality control tests for oral mucosal epithelial cell sheets.

**Additional file 4: Figure S1. **Fabrication and quality control tests for cultured autologous oral mucosal epithelial cell sheets. Morphology of an autologous oral mucosa-derived epithelial cell sheet (left panels), morphology of oral mucosa cells before transport to the hospital where transplantation was performed (middle panels), and histogram of the percentage of epithelial cells in the cell sheet measured by flow cytometry detection of cytokeratin positive cells (right panels).

## Data Availability

The datasets used and/or analyzed during the current study are available from the corresponding author upon reasonable request.

## Abbreviations

CEA: Congenital esophageal atresia
CES: Congenital esophageal stenosis
EBD: Endoscopic balloon dilatation
ESD: Endoscopic submucosal dissection
ESCC: Esophageal squamous cell carcinoma
CPF: Cell processing facility
SOP: Standard operating procedures
*α*-SMA: Alpha-smooth muscle actin
