# Supplementary material for: Evaluation of safety and efficacy of autologous oral mucosa-derived epithelial cell sheet transplantation for prevention of anastomotic restenosis in congenital esophageal atresia and congenital esophageal stenosis
Source: Stem Cell Res Ther. 2023 Apr 13;14:86. doi: 10.1186/s13287-023-03321-8 (PMC10099682; doi:10.1186/s13287-023-03321-8)
Supplement: Supplementary file 1 — Additional file 1: Table S1. Preparation of oral mucosal epithelial cell sheets. [file 13287_2023_3321_MOESM1_ESM.pdf]

Supplemental Table 1. Preparation of oral mucosal epithelial cell sheets

		Cell sheet ID		
		ESC-002	ESC-003	ESC-004
Size of mucosal tissue	Major axis	18.00 mm	10.94 mm	17.00 mm
	Minor axis	10.00 mm	7.19 mm	8.33 mm
	Area	1.413 cm^2	0.618 cm^2	1.111 cm^2
Total number of cells/tissue		3.25×10^6 cells	1.55×10^6 cells	1.46×10^6 cells
Number of cells/cm^2		2.30×10^6 cells	2.50×10^6 cells	1.31×10^6 cells
Viability		97.04 %	97.17 %	85.84 %
Seeding density/insert		5.00×10^5 cells	5.00×10^5 cells	3.60×10^5 cells
Number of cell sheets prepared		6	3	4
Number of cell sheets used for transplantation		4	3	3
