# Supplementary material for: Evaluation of safety and efficacy of autologous oral mucosa-derived epithelial cell sheet transplantation for prevention of anastomotic restenosis in congenital esophageal atresia and congenital esophageal stenosis
Source: Stem Cell Res Ther. 2023 Apr 13;14:86. doi: 10.1186/s13287-023-03321-8 (PMC10099682; doi:10.1186/s13287-023-03321-8)
Supplement: Supplementary file 2 — Additional file 2: Table S2. Inspection items for epithelial cell sheet transplantation and their timing. [file 13287_2023_3321_MOESM2_ESM.pdf]

Observation/evaluation date		Before treatment	Cell collection	Operation date	Day 1 after operation	Week 1	Week 3	Week 4	Week 12	Week 24	Week 36	Week 48	Termination
Acceptable advance/delay in schedule		4 weeks in advance of operation date	2 weeks in advance of operation date	0 days	1 day	±2 days	±1 week	±1 week	±4 weeks	±4 weeks	±4 weeks	±4 weeks	-
Informed consent		○, ○ (Collection)		○ (Transplantation)									
Collection of blood and oral mucosa			○										
Clinical condition (general)	Vital signs	○		○	○	○	○	○	○	○	○	○	○
Clinical condition (stenosis)	Difficulty in swallowing, food stuck	○		○	○	○	○	○	○	○	○	○	○
Clinical examination	Blood test	○				○	(○)	○	○			○	○
	X-ray	○				○		○	○			○	○
Endoscopic examination	Presence of stenosis	○		○		○		○	○			○	○
	Sheet fixing, epithelialization			○		○		○	○			○	○
Esophagography	Presence of stenosis	○						○	○			○	○
Safety	Adverse event	↔											

Supplemental Table 2. Inspection items for epithelial cell sheet transplantation and their timing
