# Supplementary material for: Evaluation of safety and efficacy of autologous oral mucosa-derived epithelial cell sheet transplantation for prevention of anastomotic restenosis in congenital esophageal atresia and congenital esophageal stenosis
Source: Stem Cell Res Ther. 2023 Apr 13;14:86. doi: 10.1186/s13287-023-03321-8 (PMC10099682; doi:10.1186/s13287-023-03321-8)
Supplement: Supplementary file 3 — Additional file 3: Table S3. Quality control tests for oral mucosal epithelial cell sheets. [file 13287_2023_3321_MOESM3_ESM.pdf]

Supplemental Table 3. Quality control tests for oral mucosal epithelial cell sheets

	Criteria	Results		
		ESC002	ESC003	ESC004
Sterility tests				
Aerobic bacteria	(-)	(-)	(-)	(-)
Anaerobic bacteria	(-)	(-)	(-)	(-)
Fungi	(-)	(-)	(-)	(-)
Endotoxin test	<1.0EU/mL	<0.15EU/mL	<0.15EU/mL	<0.15EU/mL
Mycoplasma tests				
Nucleic acid amplification test (NAT)	(-)	(-)	(-)	(-)
Quality of the sheet				
Total cells	>1.0×10^5 cells/sheet	1.42×10^6 cells/sheet	2.23×10^6 cells/sheet	1.37×10^6 cells/sheet
Cellular density	>2.38×10^4 cell/cm^2	3.38×10^5 cell/cm^2	5.31×10^5 cell/cm^2	3.26×10^5 cell/cm^2
Viability	>70%	98.0%	99.6%	98.1%
Percentage of epithelial cells	>70%	98.1%	99.7%	99.2%
