# Supplementary figures and images for: Evaluation of safety and efficacy of autologous oral mucosa-derived epithelial cell sheet transplantation for prevention of anastomotic restenosis in congenital esophageal atresia and congenital esophageal stenosis
Source: Stem Cell Res Ther. 2023 Apr 13;14:86. doi: 10.1186/s13287-023-03321-8 (PMC10099682; doi:10.1186/s13287-023-03321-8)

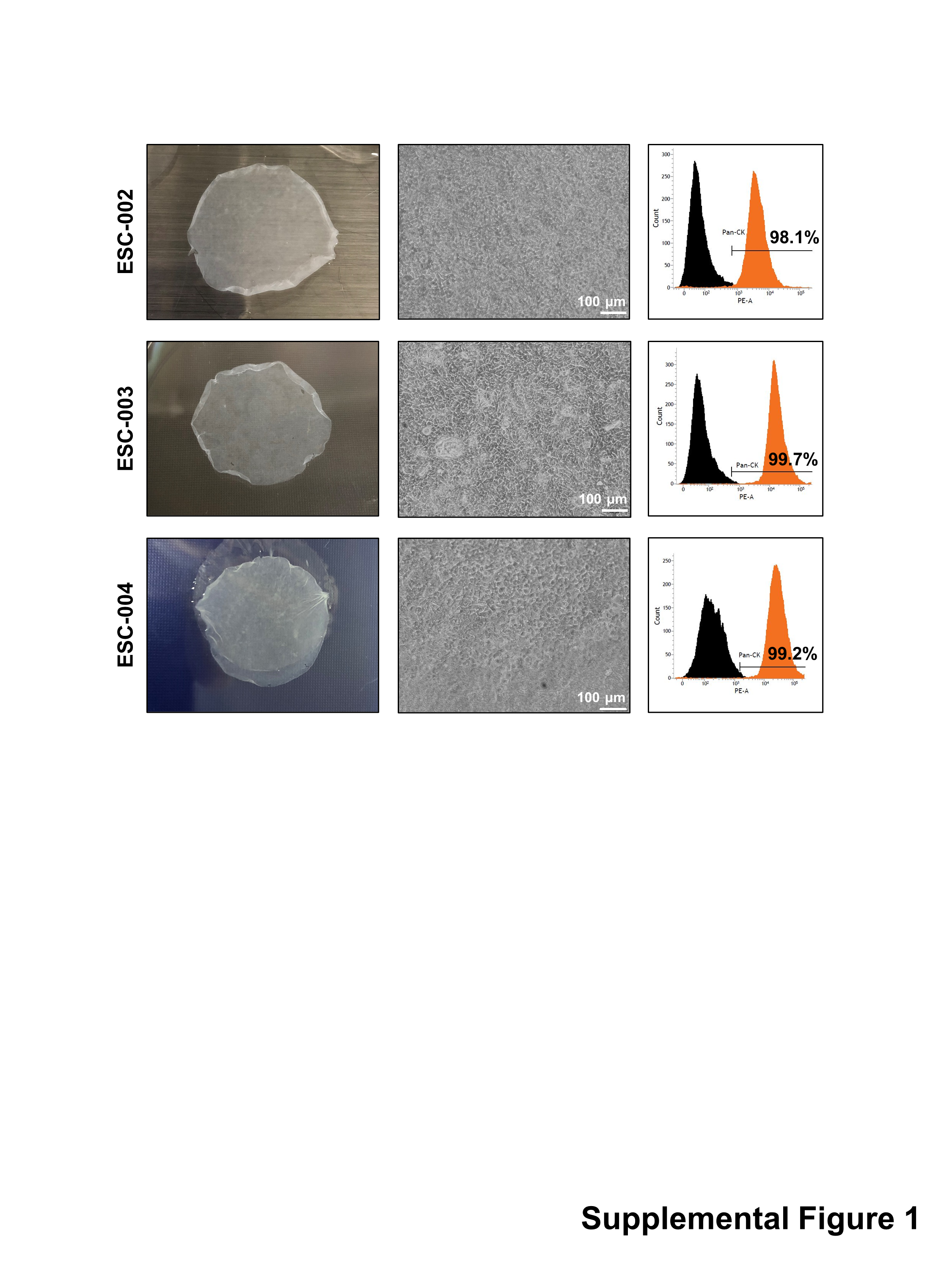

Supplement: Supplementary file 4 — Additional file 4: Figure S1. Fabrication and quality control tests for cultured autologous oral mucosal epithelial cell sheets. Morphology of an autologous oral mucosa-derived epithelial cell sheet (left panels), morphology of oral mucosa cells before transport to the hospital where transplantation was performed (middle panels), and histogram of the percentage of epithelial cells in the cell sheet measured by flow cytometry detection of cytokeratin positive cells (right panels). [file 13287_2023_3321_MOESM4_ESM.tif]
